# Hemoglobin Measurement by Point-of-Care Blood Gas Analysis Versus Central Laboratory in Hemodialysis Patients

**DOI:** 10.3390/jcm14176220

**Published:** 2025-09-03

**Authors:** Haris Omić, Michael Eder, Simon Hoffmann, Daniela Gerges

**Affiliations:** Division of Nephrology and Dialysis, Department of Medicine III, Medical University of Vienna, 1090 Vienna, Austria

**Keywords:** hemodialysis, anemia, hemoglobin, blood gas analysis

## Abstract

**Background:** In hemodialysis patients, precise hemoglobin (Hb) monitoring is essential for anemia management. Point-of-care blood gas analyzers (BGAs), such as the ABL800 Flex, offer rapid Hb determinations, but their accordance and comparability with central laboratory measurements remains to be assessed in the hemodialysis setting. **Methods:** We performed a retrospective analysis (April 2017–February 2024) of 10,802 paired Hb measurements from 291 hemodialysis patients. BGA and laboratory values within 90 min were compared using paired *t*-tests, non-inferiority testing (margin 0.5 g/dL), a Bland–Altman analysis, and linear regression. **Results:** The mean ± standard deviation Hb (g/dL) values were 10.14 ± 1.64 (BGA) versus 9.90 ± 1.55 (laboratory). The overall mean difference (BGA—laboratory) was 0.24 ± 0.49 g/dL (95% CI: 0.23–0.25), demonstrating non-inferiority (*p* < 0.0001). Measurement delay correlated with increasing analysis discrepancies (mean difference in g/dL: 0.22 at <30 min vs. 0.27 at 60–90 min; *p* < 0.001). We derived the equation of laboratory Hb = 0.90 × BGA Hb + 0.72; a simplified correction (BGA−0.3 g/dL) produced a mean absolute error (MAE) of 0.30 g/dL and root mean square error (RMSE) of 0.50 g/dL, and patient-level 10-fold cross-validation yielded MAE ≈ 0.30 and RMSE ≈ 0.49 g/dL. The Bland–Altman analysis confirmed a small systematic bias of 0.24 g/dL with 95% limits of agreement ranging from −0.73 to +1.21 g/dL. Conclusions: BGA Hb measurements via the ABL800 Flex are non-inferior to central laboratory values across clinical scenarios, with minimal bias. After regression correction, the estimated total error was ≈0.78 g/dL. If hemodialysis centers accept this level of total error and apply confirmatory testing near decision points, BGA could be used to guide anemia management.

## 1. Introduction

Chronic kidney disease (CKD) and its progression to end-stage renal disease (ESRD) necessitate the implementation of hemodialysis, a life-sustaining treatment that filters waste products and excess fluid from the blood [1]. In this context, accurate and timely monitoring of hemoglobin (Hb) levels is paramount to managing anemia, a common complication among dialysis patients [2]. The assessment of Hb concentrations in dialysis patients is not merely a routine test but a critical element of patient care [3]. It guides the administration of erythropoiesis-stimulating agents (ESAs) and iron therapy, directly impacting patient well-being and quality of life [4]. Anemia in dialysis patients is multifactorial, attributed to the decreased production of erythropoietin by the failing kidneys, the shortened lifespan of red blood cells, and other factors such as nutrient deficiencies and inflammation [5].

Blood gas analyzers (BGAs), commonly utilized for rapid assessment of blood gases, electrolytes, and metabolites in critical care settings, also offer the advantage of immediate Hb determination [6,7]. The use of BGA analyses in hemodialysis units is of particular interest due to the potential for real-time clinical decision-making. This immediacy can be crucial during dialysis sessions, allowing for on-the-spot adjustments to treatment protocols based on the patient’s current hemodynamic status [8].

However, the reliability and accuracy of Hb measurements obtained from BGAs as compared to those from central laboratory testing remain subjects of debate [9,10,11,12,13]. Central laboratory testing is typically considered the gold standard and employs methodologies such as automated hematology analyzers that provide precise and comprehensive Hb evaluations. While accurate, these tests require venous blood samples, longer processing times, and are resource-intensive, which may delay clinical decisions in the dialysis setting [14]. The primary aim of this study was to assess whether point-of-care Hb measurements obtained during routine hemodialysis sessions using a BGA are sufficiently accurate and precise compared with central laboratory Hb measurements to guide real-time clinical decisions. Although many dialysis patients undergo routine laboratory testing, the timeliness of central laboratory results varies substantially (turnaround times up to 180 min for hematology results) and may not always support immediate, intradialytic decisions [15]. In contrast, blood gas analyzers and other POCT devices can provide Hb results within minutes, enabling more rapid clinical action [16]. Rapid, reliable bedside Hb testing could, therefore, have particular value in settings where laboratory turnaround is prolonged, where dialysis facilities operate remotely from a hospital laboratory, or where transport and processing delays are common, including night shifts and low-resource regions.

We hypothesized that BGA Hb monitoring via the ABL Flex 800 is non-inferior to central laboratory measurements (non-inferiority margin 0.5 g/dL) and sought to derive a simple correction formula for clinical application that may be used for anemia management, especially in out-of-hospital and resource-limited settings.

## 2. Methods

This study was conducted as a retrospective analysis of a large cohort of dialysis patients treated at the Medical University of Vienna, Division of Nephrology and Dialysis from 1 April 2017 to 1 February 2024. The data collection focused on Hb measurements obtained through two distinct methods: a BGA analysis using the ABL800 Flex system (Radiometer Medical ApS, Copenhagen, Denmark) and standard laboratory testing. In our study, we analyzed 164,085 Hb tests conducted through both BGA and standard laboratory methods. To ensure the accuracy and reliability of our data, we focused exclusively on laboratory results obtained within a 90 min window between measurements, including only the most stable and representative samples. The study was approved by the Institutional Review Board of the Medical University of Vienna (EC-Nr: 2312/2024).

### 2.1. Blood Sampling

BGA (point of-care-testing; POCT) testing was conducted using an ABL800 Flex standard blood sampler, with a capacity of 1.7 mL, manufactured by Radiometer Medical ApS, Copenhagen, Denmark. The blood sample was collected during the dialysis session and was transferred from the syringe to the analyzer. It then underwent hemolysis by exposure to ultrasound at 30 kHz, using one microliter of the blood. The Hb level was measured using a spectrophotometric method, which involves passing light through the sample at 128 distinct wavelengths ranging from 478 to 672 nm. This light was channeled through glass-fiber optics and separated into the individual wavelengths by diffraction grating. The measurement was captured by an array of 128 photodiodes. The Hb in the blood sample was then calculated based on the Lambert–Beer law.

In the laboratory, Hb quantification was performed using the sodium lauryl sulfate (SLS) Hb technique on the Sysmex XN-Series analyzers (Sysmex Corporation, Kobe, Japan). The analysis commenced with the collection of 3 mL of blood treated with EDTA as an anticoagulant. The SLS method involves a series of processes beginning with a hemolytic reaction in which SLS attaches to the erythrocyte membrane through both ionic and hydrophobic bonds, leading to the release of Hb. Subsequent steps include the modification of the globin molecule structure by SLS, and the oxidation of heme iron from its divalent to trivalent form, a process aided by oxygen. Finally, the Hb levels are determined by the absorption of light at a wavelength of 555 nm. Analytical performances for the point-of-care blood gas analyzer and the central laboratory analyzer are summarized from manufacturer performance data and instrument reproducibility studies. The radiometer (ABL Flex series, by Radiometer Medical ApS, Copenhagen, Denmark) reports 95% confidence intervals for total hemoglobin (tHb) at multiple control levels; for example, manufacturer-reported 95% CIs (and acceptance criteria) include 7 g/dL (−0.01 to +0.11; acceptance ±0.3), 15 g/dL (+0.29 to +0.44; acceptance ±0.5), and 25 g/dL (+0.90 to +1.23; acceptance ±1.3). Sysmex XN manufacturer (Sysmex Corporation, Kobe, Japan) reproducibility data report Hb performances by control level at ~5.90 g/dL (level 1), total SD 0.072 g/dL (total CV 1.22%); at ~12.02 g/dL (level 2), total SD 0.149 g/dL (total CV 1.24%); and at ~16.34 g/dL (level 3), total SD 0.220 g/dL (total CV 1.35%). Instruments were maintained under routine laboratory QC and calibration procedures per institutional policy.

### 2.2. Statistical Analyses

The initial analysis included descriptive statistics to summarize the data, including age, means of Hb, standard deviations (SD), and ranges for Hb values obtained from both BGA and laboratory methods. To assess the variability of Hb levels across the defined categories and between the two testing methods, an ANOVA was conducted. We stratified the Hb data into several separate categories to conduct a more granular analysis of how varying Hb levels might influence the results. Those were defined according to the central laboratory Hb values, which were considered the reference standard for categorization. These categories were designed to encompass a range of Hb concentrations, specifically: 6–7 g/dL, 7.1–8 g/dL, 8.1–9 g/dL, 9.1–10 g/dL, 10.1–11 g/dL, and greater than 11 g/dL (categories 1–6). For each category, paired *t*-tests were used to compare the mean Hb levels measured by BGA and laboratory.

Additionally, to assess age-related variations, we divided the study population into three age groups: 20–40 years, 41–60 years, and over 60 years. Additionally, delay between BGA and laboratory measurements were stratified. For each paired observation, we defined measurement delay as the difference between the start times of the two measurement processes: the time at which the bedside BGA measurement was started and the time at which the central-laboratory measurement process for the paired sample began (recorded as the laboratory arrival/analysis timestamp). The non-inferiority margin for comparing measurement methods was set at 0.5 g/dL, slightly lower than previously proposed [17]. The non-inferiority margin of ±0.5 g/dL (±5 g/L) was chosen a priori on clinical grounds and is consistent with prior evaluations of point-of-care Hb devices, which have used ±0.5 g/dL and ≈±5% relative differences as clinically relevant thresholds for transfusion and anemia-management decisions [18,19]. At a cohort mean Hb of approximately 10 g/dL, ±0.5 g/dL corresponds to ±5% and is comparable to commonly accepted device performance thresholds. Because regulatory total allowable error criteria combine both bias and imprecision, we separately report and compare both the mean bias (and its confidence interval) and the Bland–Altman limits of agreement to reflect systematic and random components of disagreement. A Kruskal–Wallis test was used to analyze the effects of measurement delays between BGA and laboratory measurements on the Hb values. The linear regression was utilized to deliver a formula to correct the BGA values to standard laboratory results. Internal validation used patient-level grouped 10-fold cross-validation to avoid optimistic bias from repeated measures within patients; all pairs from the same patient were assigned to the same fold. As patients contributed different numbers of paired measurements, fold test-set row counts varied; therefore, we reported both the unweighted mean (±SD) across folds and the fold-weighted mean (weighted by number of test-set pairs) as the dataset-level estimates. We additionally performed a patient-level 70/30 split and a clustered (patient) bootstrap to estimate uncertainty. Agreement between paired BGA and central laboratory Hb results was assessed using a Bland–Altman analysis. For each pair, the difference was plotted against the mean of the two methods. The overall bias was calculated as the mean of these differences, and the SD of the differences was determined. Ninety-five percent limits of agreement (LoAs) were defined as bias ± 1.96 × SD and displayed using the Bland–Altman plot. A statistical analysis was performed using the commercially available IBM SPSS Statistics software (version 29.0.2.0. for Mac) and GraphPad Prism (GraphPad Prism 10.0.3(217) Macintosh Version by Software MacKiev© 1994–2023 GraphPad Software, LLC).

## 3. Results

**Figure 1:**
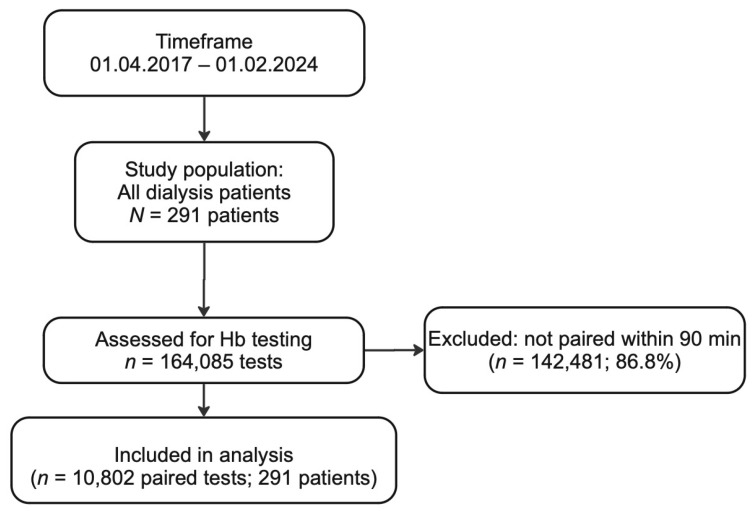
Flow chart for the selection process for paired hemoglobin measurements included in our analysis. Over a seven-year period (1 April 2017–1 February 2024), a total of 164,085 hemoglobin (Hb) tests were performed in our hemodialysis cohort. Of these, 142,481 tests (86.8%) were excluded because the corresponding blood gas analyzer (BGA) and central laboratory samples were measured more than 90 min apart from one another. The remaining 10,802 paired tests (6.6%) from 291 unique patients met the inclusion criterion of having both BGA and laboratory hemoglobin results within a 90 min window. These 10,802 paired measurements were then subjected to comparative and agreement analyses.

Between 2017 and 2024, the total of 21,604 (10,802 pairs) measurements from 291 patients were analyzed to compare BGA monitoring with laboratory Hb testing (Figure 1). The study analyzed data from participants with an average age of 63.4 years (SD = 15.4), ranging from 23 to 93 years. The mean Hb levels were 9.90 (SD = 1.55) for the laboratory and 10.14 (SD = 1.64) for the BGA. The overall Hb levels measured by the laboratory ranged from 6.57 g/dL to 11.9 g/dL, while BGA measurements varied from 6.84 g/dL to 12.2 g/dL (Table 1).

The overall mean difference between BGA and laboratory measurements was 0.24 (SD = 0.49), with 95% confidence intervals ranging from 0.25 to 0.23. However, this difference was not clinically significant, as evidenced by the non-inferiority test, which confirmed that BGA testing is non-inferior to laboratory analyses, with a margin of 0.5 g/dL (*p* < 0.0001). For the age group of 20–40 years, the mean difference was 0.26 g/dL (SD = 0.40). In the 41–60 years age group, the mean difference was 0.25 g/dL (SD = 0.48), and in the >60 years group it was 0.23 (SD = 0.52), with no statistically significant discrepancy between BGA and laboratory measurements across different age groups (*p* = 0.34).

### 3.1. Category-Specific Hemoglobin Assessment

The analysis of Hb measurements across predefined categories revealed distinct mean values for both BGA and standard laboratory testing. In category 1, the mean laboratory Hb was 6.57 g/dL (SD = 0.25) compared to the BGA’s 6.84 g/dL (SD = 0.50). Category 2 showed a laboratory Hb of 7.52 g/dL (SD = 0.28) versus the BGA’s 7.74 g/dL (SD = 0.50). This pattern continued across the categories, with category 3 displaying laboratory Hb at 8.49 g/dL (SD = 0.28) and the BGA at 8.73 g/dL (SD = 0.55), category 4 with laboratory Hb at 9.47 g/dL (SD = 0.28) against the BGA at 9.69 g/dL (SD = 0.54), category 5 with laboratory Hb at 10.4 g/dL (SD = 0.28) compared to the BGA at 10.7 g/dL (SD = 0.57), and category 6 with laboratory Hb at 11.9 g/dL (SD = 0.79) relative to the BGA at 12.2 g/dL (SD = 0.95) (Table 1). In the non-inferiority analysis of BGA versus laboratory measurements across Hb categories, all evaluated categories demonstrated that BGA testing is not inferior to laboratory testing, adhering to a predetermined non-inferiority margin (0.5 g/dL). The mean differences between BGA and laboratory results ranged from 0.22 to 0.26, with category 4 exhibiting the lowest mean difference (0.22) and category 6 the highest (0.26). However, the lower bound of the 95% confidence interval for the mean differences stayed above the non-inferiority margin in all categories, affirming the non-inferiority of BGA to laboratory results. The lower confidence interval limits ranged from 0.20 in categories 1 and 2 to 0.24 in category 6.

### 3.2. Prediction of Laboratory Hemoglobin

**Figure 2:**
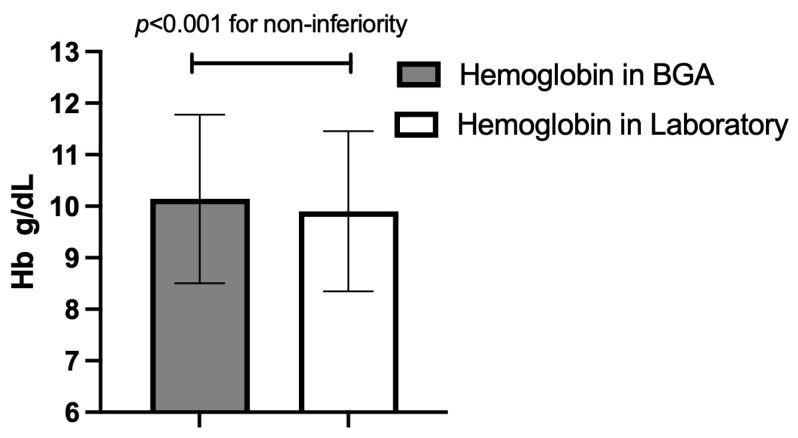
Overall mean hemoglobin (Hb) concentration (±standard deviation) for the two measurement methods across all paired samples; *p*-value for non-inferiority at a 0.5 g/dL margin. BGA: blood gas analyzer.

**Figure 3:**
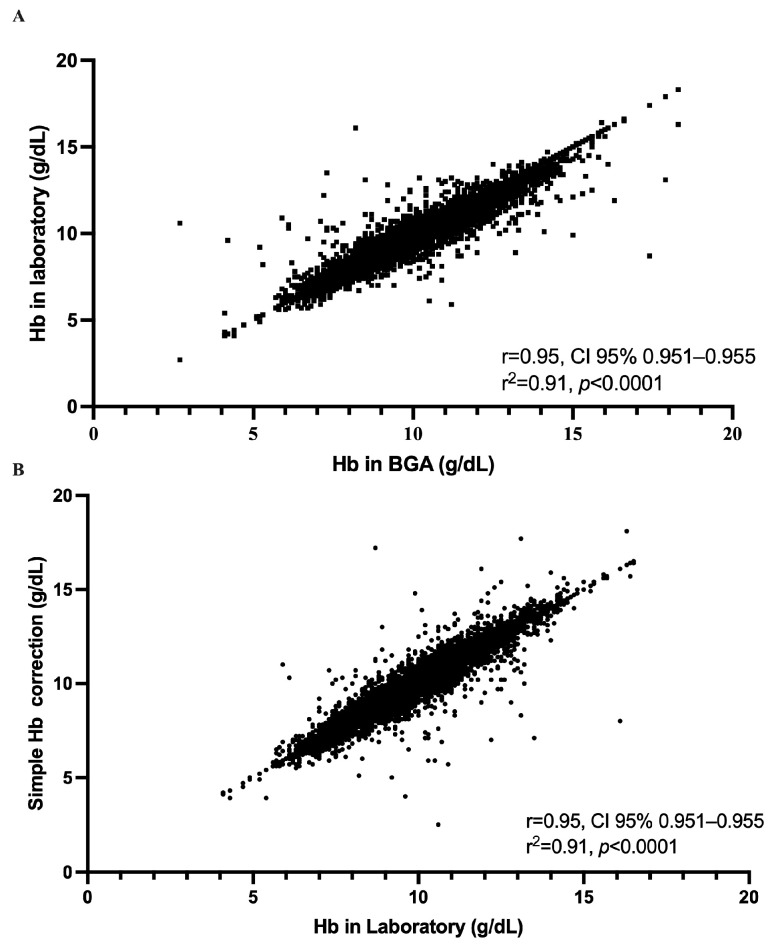
Strength of the relationship between hemoglobin measurements obtained by the ABL800 Flex blood gas analyzer (BGA) and those obtained in the central laboratory, both before and after application of our simplified correction. (**A**) Each of the 10,802 paired measurements is plotted with the raw BGA hemoglobin concentration on the *x*-axis and the corresponding laboratory value on the *y*-axis. The data form a tightly clustered cloud extending from approximately 5 to 17 g/dL, and linear regression yields a Pearson correlation coefficient of r = 0.95 (95% CI, 0.95–0.95) with R^2^ = 0.91 (*p* < 0.0001). (**B**) The effect of applying our simple correction, subtracting 0.3 g/dL from each BGA result, by plotting the corrected BGA values against the laboratory measurements. The resulting scatter of points again spans roughly 5 to 17 g/dL and retains the same high degree of concordance, with r = 0.95 (95% CI, 0.95–0.95), R^2^ = 0.91, and *p* < 0.0001.

Utilizing linear regression, we derived a formula that shows a strong linear relationship between BGA and laboratory values. The regression equation [predicted laboratory Hb (g/dL) = 0.91 × BGA (g/dL) + 0.72] serves as the basis for our correction formula. The slope of the regression line, 0.91, indicates that for each unit increase in the BGA Hb value, the laboratory Hb value is expected to increase by approximately 0.91 units. The statistical analysis revealed a high correlation coefficient of 0.95, suggesting that the predicted laboratory values closely align with the actual laboratory measurements across the patient dataset (Hb for both 9.90 g/dL, Figure 2 and Figure 3).

**Figure 4:**
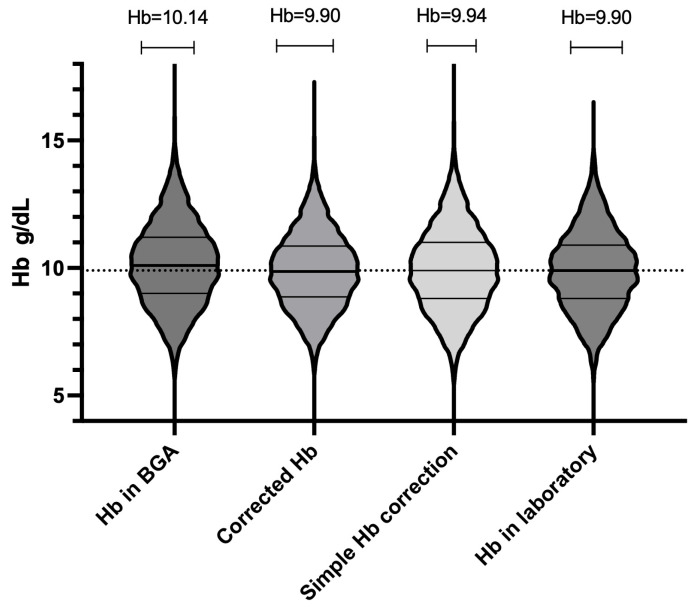
Overall distribution of hemoglobin (Hb) values for all 10,802 paired measurements (n = 291 patients) using four methods: laboratory, raw blood gas analyzer (BGA), BGA corrected by regression formula, and BGA corrected by simplified “−0.3 g/dL” method. Each violin’s width at a given Hb concentration reflects the relative density (frequency) of measurements at that value. Within each violin, the solid horizontal line marks the median. The upper and lower horizontal lines denote the 75th and 25th percentiles (interquartile range, IQR). All four violins span approximately 6.5 to 12.5 g/dL, showing substantial overlap.

**Figure 5:**
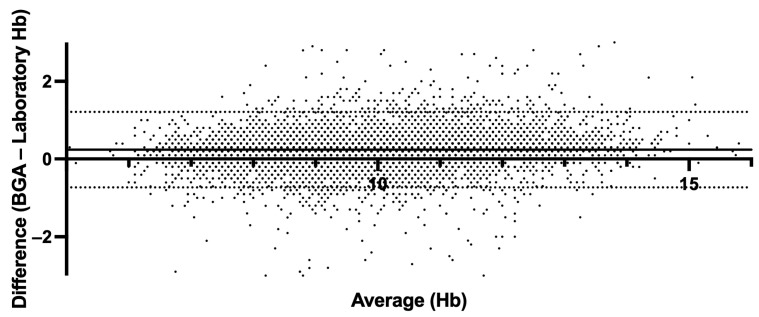
Bland–Altman plot comparing hemoglobin derived from BGA and central laboratory hemoglobin measurements. The solid line indicates mean bias (0.24 g/dL); dashed lines indicate the 95% limits of agreement (−0.73 g/dL, +1.21 g/dL).

The mean absolute error (MAE) of 0.29 and the root mean square error (RMSE) of 0.47 further attest to the accuracy of the formula. The simplified correction formula for estimating Hb values from BGA measurements (estimated laboratory Hb = BGA Hb − 0.3) has demonstrated notable accuracy and practicality in clinical application. Our analysis provides a mean estimated Hb value of 9.94 g/dL using the simplified approach, aligning with the 9.90 g/dL obtained through the more complex regression-based method and the same in the standard laboratory analysis (Hb = 9.90 g/dL), slightly lower than the direct BGA measurement average of 10.14 g/dL (Figure 4). In order to assess the agreement level between BGA and central laboratory Hb measurements, we performed a Bland–Altman analysis (Figure 5). The mean bias (BGA vs. laboratory) was 0.24 g/dL, with a standard deviation of bias of 0.49 g/dL. The 95% LoAs ranged from −0.73 g/dL to +1.2 g/dL. The mean bias of 0.24 g/dL lay well below our pre-specified non-inferiority margin of 0.5 g/dL.

### 3.3. Delay Analysis

**Figure 6:**
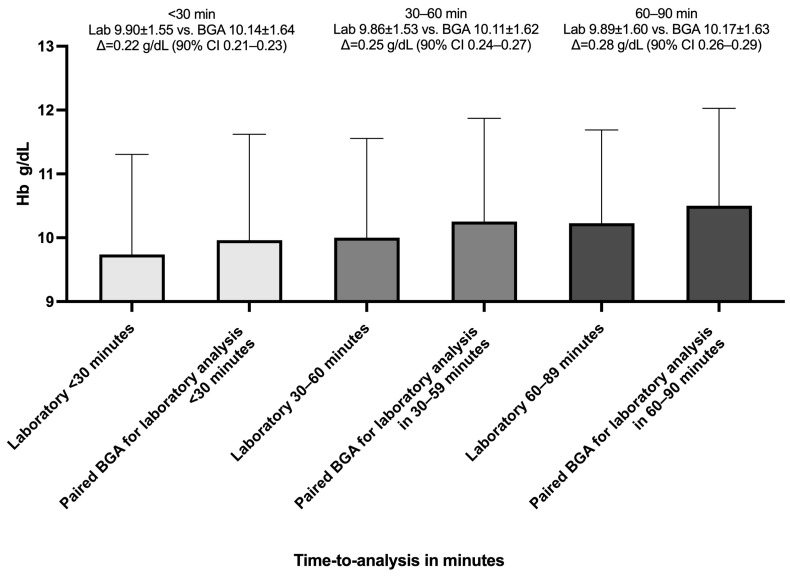
Mean hemoglobin values by measurement method and analysis delay. The bar chart shows the mean hemoglobin (Hb) concentrations measured by the central laboratory (Lab) and by the blood gas analyzer (BGA) across three time-to-central-laboratory-analysis-delay intervals: <30 min, 30–60 min, and 60–90 min. Bars are grouped by delay category. Error bars represent standard deviation; Δ represents the mean paired difference (BGA—Lab); 90% CI(Δ) ⊂ ±0.5 g/dL ⇒ equivalent (95% confidence).

The delay between BGA and laboratory measurements was categorized into intervals of under 30 min with a mean of 18 min (SD = 8.65), 30 to 60 min with a mean of 43.9 min (SD = 8.65), and 60 to 90 min with a mean of 75.4 min (SD = 8.60). Additionally, we divided the 10,802 paired Hb measurements into three categories by time between BGA and laboratory timestamps to test for equivalence (±0.5 g/dL). For paired samples analyzed within 30 min, the mean bias was 0.22 g/dL (SD 0.49), with a 90% confidence interval of 0.21 to 0.23 g/dL (Figure 6). For the 30–60-min group, the mean bias increased modestly to 0.25 g/dL (SD 0.51), with a 90% CI of 0.24 to 0.27 g/dL, again fully contained within the bounds. In the 60–90-min category, the mean bias was 0.28 g/dL (SD 0.47), and the 90% CI values ranged from 0.26 to 0.29 g/dL.

### 3.4. Rapid Analysis (Processing Times “<5” and “<10” Minutes)

**Figure 7:**
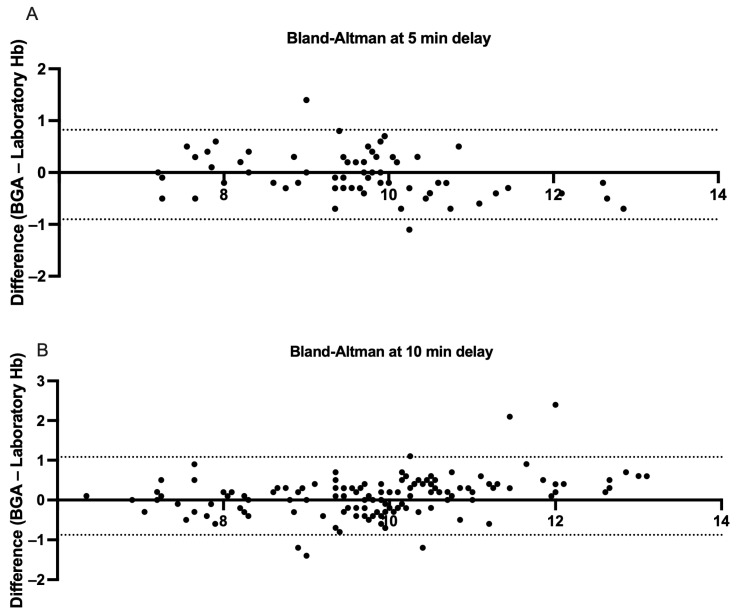
Bland–Altman measurements comparing hemoglobin samples derived from BGA and central laboratory hemoglobin measurements stratified by <5 and <10 min central laboratory analysis delays. (**A**) Each point represents a paired hemoglobin measurement analyzed in the central laboratory within five minutes of sampling or BGA testing (n = 66 pairs). The *x*-axis shows the mean of the BGA and laboratory values for each pair, and the *y*-axis displays the difference (BGA—laboratory). The dashed lines mark the 95% limits of agreement (−0.90 to +0.82 g/dL), showing that 95% of individual differences lie within this range. (**B**) The analogous analysis for pairs tested within ten minutes of sampling or BGA analysis (n = 133 pairs). The 95% limits of agreement (−0.87 to +1.01 g/dL) are indicated by the dashed lines.

To further assess the effect of processing delay on agreement between BGA and central laboratory Hb measurements, we analyzed two rapid-turnaround subgroups: pairs measured within 5 min (n = 66) and within 10 min (n = 133) of each other. In the ≤5 min subgroup, the mean paired difference was −0.04 g/dL (SD 0.44), with a two-tailed paired *t*-test confirming no significant difference between methods (95% CI −0.07 to +0.15 *p* = 0.47; r = 0.95, *p* < 0.0001). The Bland–Altman analysis yielded a mean bias of −0.04 g/dL and 95% LoAs ranging from −0.90 to +0.82 g/dL (Figure 7, Panel A). In the ≤10 min subgroup, the mean was +0.10 g/dL (SD 0.5), and a paired *t*-test indicated a small but statistically significant difference (95% CI −0.19 to −0.017 g/dL *p* = 0.019, r = 0.94, *p* < 0.0001). The Bland–Altman LoAs were −0.87 to +1.08 g/dL around the 0.10 g/dL bias (Figure 7, Panel B). Therefore, reducing the analysis-to-analysis interval to ≤5 min essentially eliminates systematic bias, whereas extending delays to 10 min introduces a small but measurable overestimation by the BGA.

### 3.5. Internal Validation

Using all paired records (n = 10,802 pairs from 291 patients), grouped patient-level 10-fold cross-validation produced an unweighted mean MAE of 0.30 ± 0.02 g/dL and an unweighted mean RMSE of 0.49 ± 0.06 g/dL across folds. When fold results were weighted by number of test-set pairs, the MAE was stable at 0.30 g/dL, as well as the RMSE at 0.49 g/dL. Per-fold MAE values ranged from 0.28 to 0.33 g/dL and per-fold RMSE values from 0.43 to 0.62 g/dL (see Supplementary Table S1). These grouped validation results indicate that the regression correction generalizes well across patients, with an expected average prediction error of ≈0.3 g/dL. On patient-level 70/30 validation (train pairs = 6919; test pairs = 3883), the regression model predicting laboratory Hb results from BGA Hb measurements achieved a test MAE of 0.31 g/dL and RMSE of 0.52 g/dL. The mean residual on the held-out test set was negligible (close to zero), indicating little systematic bias in out-of-sample predictions; consequently, the SD of test residuals is well approximated by the RMSE (≈0.52 g/dL). Using the practical total-error formula TE = |bias| + z × SD and assuming negligible bias, the estimated TE is ≈0.87 g/dL when z = 1.65 (one-sided 95% criterion).

## 4. Discussion

Between April 2017 and February 2024, we retrospectively examined 21,604 paired Hb determinations obtained from 291 hemodialysis patients to compare point-of-care BGA measurements with central laboratory results. Central to the discussion is the observed mean Hb level discrepancy between BGA (10.14 g/dL) and laboratory (9.90 g/dL) methods. The derived overall mean difference of 0.24 g/dL, supported by a statistically robust non-inferiority analysis, underscores the BGA’s reliability compared to traditional laboratory methods, within a clinically acceptable variance margin.

Furtherly, the age-specific analysis showcased minimal variance across different demographic segments, fortifying the assertion of the BGA’s consistent performance. The absence of significant discrepancy across age groups, as indicated by the non-significant *p*-value (0.34), enhances the argument for the BGA’s broader clinical utility.

Category-specific evaluations further illuminate the relative stability in Hb readings across varying Hb ranges. The slight differences noted between BGA and laboratory readings across Hb categories, and the maintained non-inferiority across these spectra, provide a granular understanding of the measurement dynamics. Leveraging this large and longitudinal dataset, we were able to characterize both the systematic bias and the LoA between the two methods across clinically relevant time delays. Our analysis demonstrated that despite a modest positive bias of approximately 0.24 g/dL in raw BGA values, the vast majority of individual differences remained within our pre-specified ±0.5 g/dL equivalence margin, even when sample analysis was delayed by up to 90 min.

The incremental increase in discrepancy over time, especially noted beyond the 30 min mark, signifies a potential temporal impact on BGA accuracy. This time-dependent variability necessitates a timelier analysis post-blood draw to minimize potential discrepancies in clinical interpretation. Nonetheless, whether the modest discrepancies we observed might translate into clinically meaningful differences in anemia management remains to be determined. Several non-dialysis studies have demonstrated strong agreement between BGA and central laboratory Hb measurements in emergency, perioperative, and laboratory settings [20,21,22]. Recent work in COPD patients found arterial blood gas Hb measurements to differ by <0.3 g/dL from venous laboratory values, with >95% correlation [23].

However, not all investigations have reached the same conclusion. In a prospective comparison of the Roche AVL OMNI S blood gas analyzer and the hospital central laboratory results, BGA-derived Hb, hematocrit, sodium (Na+), and potassium (K+) values were systematically lower than their laboratory counterparts (*p* < 0.0001), and nearly 30% of all measurements exceeded the US-CLIA allowable total error limits, even though the mean biases for Hb, Na^+^, and K^+^ individually fell within the specified cut-offs [24]. Similarly, a retrospective cross-sectional study of 1927 paired samples demonstrated unacceptably wide Bland–Altman LoAs (e.g., −5.0 to +4.0 g/dL for Hb) and Cohen’s κ values ≤0.60, leading the authors to conclude that BGA and laboratory hematology results could not be used interchangeably [25].

Several factors may account for these discordant findings. First, differences in analyzer technology, including the number and selection of wavelengths used for spectrophotometry and the specific lysing agents (e.g., SLS vs. ultrasonic hemolysis), can introduce systematic measurement shifts. Second, pre-analytical variables such as the sample source (arterial versus venous), anticoagulant type, and site of blood draw (e.g., direct from the dialysis circuit versus a central vascular access) can affect the Hb concentration through localized hemolysis. Third, variations in instrument maintenance, calibration schedules, and quality control procedures between point-of-care and central laboratory settings may further widen the gap. On the other hand, the dialysis-specific factors, such as intradialytic fluid shifts, access recirculation, and sampling delays, could affect the BGA’s accuracy. Only limited data have compared BGA versus laboratory Hb results in hemodialysis populations [26]. Our study bridges this gap by evaluating 10,802 paired Hb measurements over seven years in a large dialysis cohort. Our analysis aimed to establish a reliable correction formula that enables clinicians to predict standard laboratory Hb values from BGA measurements.

Our correction formula (Hb = BGA − 0.3), juxtaposed against the more complex regression-based equation, stands out for its clinical expediency without a significant compromise on accuracy. The mean Hb estimation via this simplified method (9.94 g/dL) closely mirrors the laboratory standard (9.90 g/dL), reinforcing its practical utility in clinical environments.

The strong correlation coefficient (0.95) alongside the minimal MAE and RMSE values in both complex and simplified formulas attests to the robustness of our predictive models. In addition, the internal validation demonstrates that the regression correction reduces the mean absolute error to ~0.3 g/dL and yields an RMSE of ~0.5 g/dL across a large, clustered dataset of 10,802 paired measurements. These errors are small relative to clinical decision thresholds (non-inferiority margin ±0.5 g/dL), but the Bland–Altman limits show that individual paired differences can be larger; therefore, the correction improves the average agreement but cannot eliminate occasional clinically meaningful mismatches. These statistical indicators not only validate the accuracy of the formulas but also underscore their potential to streamline clinical workflows by enabling rapid, near-accurate Hb estimations. Additionally, across all three sampling delay categories (<30 min, 30–60 min, and 60–90 min), the 90% confidence intervals for the mean differences of Hb values lay entirely within our pre–specified ±0.5 g/dL equivalence bounds. These results confirm that point-of-care BGA Hb measurements using ABL800 Flex are clinically interchangeable with central laboratory values, even when the analysis is delayed by up to 90 min. Although current CLIA total-allowable-error criteria are stricter than our predefined equivalence margin, we chose ±0.5 g/dL (≈±5% at Hb = 10 g/dL) because it reflects previously published POCT thresholds and clinical decision limits [27]. Using the Bland–Altman parameters after regression correction (bias ≈ 0, SD ≈ 0.47 g/dL), the estimated total error would be ≈ 0.78 g/dL, which exceeds the strict CLIA total allowable error margin (~0.4 g/dL at Hb ≈ 10 g/dL). Thus, although the mean bias is small and within our ±0.5 g/dL equivalence margin, the combined effect of residual imprecision means the corrected BGA does not meet the most stringent regulatory total error threshold. KDIGO guidelines define Hb thresholds to guide ESA initiation and dose adjustment [28]. Given that our regression-corrected BGA measurements exhibit minimal systematic bias but a non-negligible total error (~0.8–0.9 g/dL), clinical practice should balance speed with caution; corrected BGA values may be used for routine monitoring and timely intra-dialysis decisions provided individual centers accept this level of total error and implement robust POCT quality control, but any result falling within a predefined buffer zone around KDIGO treatment thresholds, or any value that would trigger major interventions, should be confirmed by the central laboratory before changing the ESA dosing. This pragmatic approach preserves the advantage of rapid point-of-care information while minimizing the risk of inappropriate ESA adjustments due to residual analytical variability.

The limitations of our study include the retrospective design and single-center setting, which may limit the generalizability to other BGA platforms. Additionally, intradialytic fluid shifts and sampling sites (AV-fistula vs. permanent dialysis catheter) were not separately analyzed, warranting prospective evaluation. Although our dataset contains 10,802 paired Hb measurements, these originate from only 291 individual patients. Repeated measures within patients introduce within-subject correlation and could reduce the effective sample size for inferences about independent observations. We addressed this by reporting patient counts alongside test counts, and by using paired analyses for method comparison. Nevertheless, we acknowledge that the large number of repeated samples from the same subjects may inflate the precision and that the results should be interpreted by considering this limitation. Additionally, we did not have a uniform laboratory hemolysis index for all samples in this retrospective dataset, so we could not exclude hemolyzed specimens systematically; therefore, hemolysis may have contributed to the observed imprecision. Finally, we did not exclude implausible or erroneous Hb values in this retrospective cohort, which may have inflated the observed imprecision and contributed to the higher estimated total error. Future studies using mixed-effects models or analyses stratified by patient would further account for intra-patient correlation. Although our regression correction markedly reduced the mean error and improved the precision within the cohort, external validation in independent populations is still required before recommending routine application.

At the end, the decision to rely on BGA testing for routine anemia management should be guided by local logistics and economics. In centers without on-site laboratory services, or where transport and processing delays routinely exceed the dialysis session or clinical need, POCT can materially shorten the time-to-result and support timely decisions; evidence suggests POCT may be cost-effective in these contexts [29].

In conclusion, this study validates the BGA’s reliability compared to laboratory measurements and offers a clinically viable tool for Hb estimation and anemia management in hemodialysis patients, if the total error of ~0.8–0.9 g/dL is clinically acceptable. The alignment of BGA measurements with laboratory readings, across diverse patient demographics and Hb categories, reinforces its clinical validity. Moreover, the introduction of a simplified correction formula and its internal validation offers a promising avenue for enhancing its clinical efficiency, allowing for real-time, accurate Hb assessments that can significantly impact patient management and outcomes. Future studies should assess the impact of BGA-guided ESA dosing on clinical outcomes and cost-effectiveness.

## Figures and Tables

**Table 1:** Overall and subgroup hemoglobin measurements and mean differences.

Subgroup	*n* Measurements	BGA Hb (g/dL) Mean ± SD	Laboratory Hb (g/dL) Mean ± SD	Mean Diff ±SD (BGA—Laboratory)	95% CI	*p*-Value
Overall	10,802	10.14 ± 1.64	9.90 ± 1.55	0.24 ± 0.49	0.23–0.25	<0.0001 *
Age 20–40 years	1230	10.23 ± 1.62	9.97 ± 1.48	0.26 ± 0.40	0.25–0.27	0.12
Age 41–60 years	4576	10.11 ± 1.58	9.86 ± 1.51	0.25 ± 0.48	0.24–0.26	0.21
Age >60 years	5026	10.12 ± 1.70	9.89 ± 1.60	0.23 ± 0.52	0.22–0.24	0.34
Hb 6–7 g/dL	181	6.84 ± 0.50	6.57 ± 0.25	0.27 ± 0.48	0.20–0.34	<0.001 *
Hb 7.1–8 g/dL	534	7.74 ± 0.50	7.52 ± 0.28	0.22 ± 0.50	0.16–0.28	<0.001 *
Hb 8.1–9 g/dL	1246	8.73 ± 0.55	8.49 ± 0.28	0.24 ± 0.53	0.20–0.28	<0.001 *
Hb 9.1–10 g/dL	2106	9.69 ± 0.54	9.47 ± 0.28	0.22 ± 0.54	0.18–0.26	<0.001 *
Hb 10.1–11 g/dL	3155	10.70 ± 0.57	10.40 ± 0.28	0.30 ± 0.57	0.25–0.35	<0.001 *
Hb >11 g/dL	2580	12.20 ± 0.95	11.90 ± 0.79	0.30 ± 0.60	0.24–0.36	<0.001 *

* All Hb level subgroups met non-inferiority criteria (0.5 g/dL margin).

## Data Availability

The datasets generated and analyzed during the current study are available from the corresponding author on reasonable request.

## Supplementary Materials

The following supporting information can be downloaded at: https://www.mdpi.com/article/10.3390/jcm14176220/s1, Supplemental Table S1: Results of patient-level grouped 10-fold cross-validation for the linear regression model predicting central laboratory hemoglobin from point-of-care BGA hemoglobin. Each fold lists the number of test-set paired measurements (n), the mean absolute error (MAE, in g/dL), the root mean squared error (RMSE, in g/dL), and the coefficient of determination (R^2^) observed on that fold’s test set. Folds were created so that all paired measurements from the same patient were assigned to the same fold; consequently, the number of test rows per fold varies because patients contributed different numbers of paired measurements.
