# Supplementary material for: Hemoglobin Measurement by Point-of-Care Blood Gas Analysis Versus Central Laboratory in Hemodialysis Patients
Source: J Clin Med. 2025 Sep 3;14(17):6220. doi: 10.3390/jcm14176220 (PMC12428970; doi:10.3390/jcm14176220)
Supplement: Supplementary file 1 [file jcm-14-06220-s001.zip › jcm-3845835-supplementary.pdf]

Fold	n (test pairs)	MAE (g/dL)	RMSE (g/dL)	R^2
fold 1	1061	0.28	0.46	0.93
fold 2	960	0.30	0.45	0.92
fold 3	949	0.33	0.62	0.86
fold 4	740	0.29	0.45	0.92
fold 5	1242	0.31	0.54	0.89
fold 6	1098	0.29	0.43	0.93
fold 7	708	0.33	0.52	0.90
fold 8	1683	0.30	0.47	0.92
fold 9	1296	0.31	0.51	0.87
fold 10	1061	0.30	0.49	0.91

Supplemental Table S1. Results of patient-level grouped 10-fold cross-validation for the linear regression model predicting central laboratory hemoglobin from point-of-care BGA hemoglobin. Each fold lists the number of test-set paired measurements (n), the mean absolute error (MAE, in g/dL), the root mean squared error (RMSE, in g/dL), and the coefficient of determination (R^2) observed on that fold's test set. Folds were created so that all paired measurements from the same patient were assigned to the same fold; consequently, the number of test rows per fold varies because patients contributed different numbers of paired measurements.
